# Lymphocyte function inhibition and exhaustion in sepsis: mechanisms and applications

**DOI:** 10.3389/fimmu.2026.1619478

**Published:** 2026-03-18

**Authors:** Yuan Yan, Jialian Wang, Chao Wang, Xingyu Tao, Peifeng Cheng, Jing Qin, Bailin Niu

**Affiliations:** Department of Intensive Care Medicine, Chongqing Emergency Medical Center, Chongqing University Central Hospital, School of Medicine, Chongqing University, Chongqing, China

**Keywords:** exosome, lymphocyte, negative costimulatory proteins, non-coding RNA, sepsis

## Abstract

Sepsis is a life-threatening organ dysfunction syndrome caused by a dysregulated host response to infection and is characterized by complex pathophysiological mechanisms in which immune dysfunction plays a central role. Among immune cells, lymphocytes are critically involved in both the progression and resolution of sepsis. Increasing evidence indicates that lymphocyte depletion, functional exhaustion, and phenotypic reprogramming are closely associated with persistent immunosuppression and adverse clinical outcomes. This review examines the major mechanisms underlying lymphocyte function inhibition in sepsis and organizes them into interconnected pathways, including inhibitory membrane receptors (such as PD-1, CTLA-4, and LAG-3), mitochondrial and endoplasmic reticulum stress–related organelle dysfunction, exosome-mediated intercellular communication, non-coding RNA regulatory networks, and cytokine-driven immune modulation. These mechanisms interact through shared intracellular signaling pathways, contributing to impaired proliferation, altered cytokine production, metabolic dysregulation, and apoptosis of lymphocytes. The clinical relevance of lymphocyte-based indicators is also discussed, including absolute lymphocyte counts, subpopulation distribution, and immune checkpoint expression, which show potential value in early risk stratification and prognostic assessment. In addition, emerging therapeutic strategies targeting immune checkpoints and immunometabolic dysfunction are summarized. These insights provide a structured understanding of lymphocyte inhibition in sepsis and offer potential directions for improving immune monitoring and developing individualized immunomodulatory interventions.

## 1 Introduction

Sepsis is characterized as a life-threatening organ dysfunction resulting from the host’s dysregulated response to infection (1). Sepsis occurs in 48.9 million patients worldwide, resulting in about 11 million fatalities annually, accounting for 19.7% of all global deaths (2). Comparative analyses show Chinese patients with sepsis and septic shock face greater disease occurrence and mortality than their counterparts in North America and Europe (3). Recent nationwide epidemiological data from China indicate a steady increase in the incidence of hospitalized sepsis between 2017 and 2019, reaching over 400 cases per 100,000 population annually. Age distribution analysis showed that elderly individuals (≥65 years) accounted for more than half of all hospitalized sepsis cases, while neonates and young children represented smaller proportions. Significant geographic variation was also observed, with spatial clustering patterns suggesting disparities in healthcare resources and socioeconomic factors (4).

**Figure 1:**
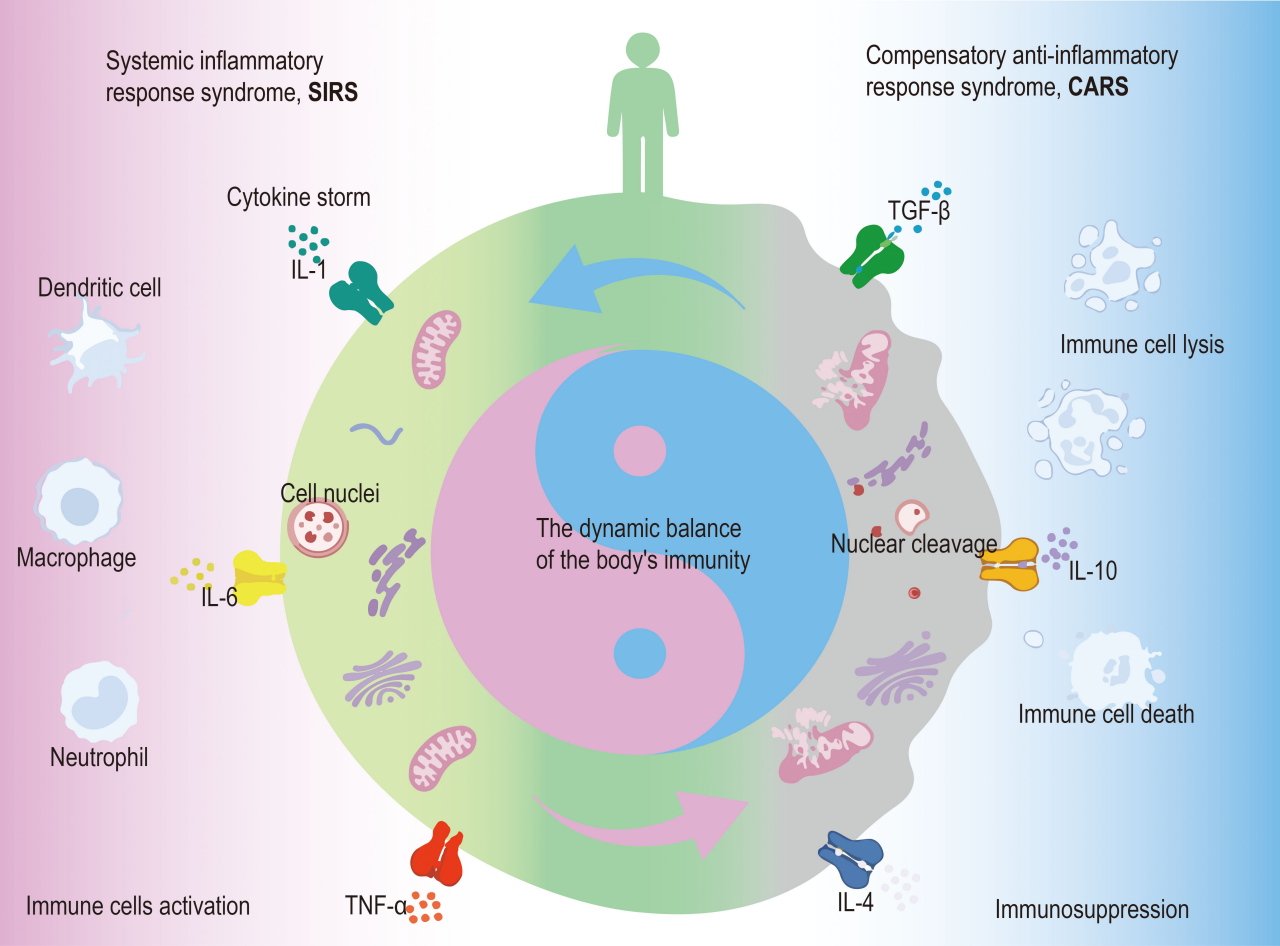
Dynamic and bidirectional immune regulation during sepsis. This schematic presents a cell-centered model of immune dysregulation in sepsis. The left side illustrates systemic inflammatory response syndrome (SIRS), characterized by activation of innate immune cells and excessive release of pro-inflammatory cytokines, including TNF-α, IL-1, and IL-6. The right side represents compensatory anti-inflammatory response syndrome (CARS), marked by increased anti-inflammatory cytokines (IL-4, IL-10, and TGF-β), immune cell dysfunction, nuclear cleavage, and cell death. The central immune cell integrates opposing inflammatory and anti-inflammatory signals, with intracellular alterations involving mitochondria and nuclear integrity. Bidirectional arrows indicate that immune activation and suppression are dynamic and reversible rather than sequential processes.

In sepsis, the body’s pro-inflammatory and anti-inflammatory immune responses are activated accordingly, namely systemic inflammatory response syndrome (SIRS) and compensatory anti-inflammatory response syndrome (CARS) (**Figure 1**). Early immune activation triggers a surge in pro-inflammatory cytokine release from innate immune cells, which are amplified in a cascade, resulting in a systemic cytokine storm that contributes to early organ dysfunction in septic patients. With advances in sepsis management strategies and improvements in integrated ICU care, early mortality associated with the hyperinflammatory phase characterized by cytokine storm has declined in many settings. However, as sepsis progresses, persistent immune dysregulation frequently develops, leading to a state of immunosuppression. This phase is characterized by increased apoptosis and functional impairment of multiple immune cell populations, including lymphocytes, macrophages, and dendritic cells. Among these, lymphocyte dysfunction is particularly prominent, manifesting as both functional inhibition and exhaustion phenotypes, and has been closely associated with adverse clinical outcomes in septic patient (5–7). Notably, the pediatric immune system is developmentally distinct from that of adults. Neonates exhibit a Th2-skewed immune profile, reduced memory T-cell pools, and altered innate–adaptive immune crosstalk, which may influence susceptibility to lymphocyte exhaustion and apoptosis during sepsis (8, 9).

Lymphocytes, while morphologically homogeneous, are functionally heterogeneous and include multiple lineages: T cells, B cells, natural killer cells, and various innate lymphoid cell populations. These cells mediate antibody production, direct cytotoxic elimination of infected/tumor cells, and immune regulation (10). Numerous studies have reported that T-cell depletion represents a hallmark of sepsis-associated immunosuppression. CD4+ T cells may undergo apoptosis in response to mechanisms such as cytokine storm, oxidative stress, and mitochondrial dysfunction. In addition, engagement of death receptor ligands (e.g., FasL) with their corresponding receptors on T cells activates downstream apoptotic signaling cascades, further contributing to lymphocyte loss (11, 12).

The depletion of B cells in sepsis primarily results from either increased apoptotic activity or inadequate T-helper cell support, with post-activation memory B cell populations being particularly vulnerable. Surviving B cells exhibit an exhausted phenotype marked by downregulated MHC-II expression and elevated IL-10 production. This depletion of memory B-cell populations contributes significantly to sepsis-associated immunosuppression (13, 14). B cells operate as crucial elements in the immune homeostasis of patients with sepsis, and it is hoped that in the future, more research will be devoted to sepsis B cell suppression.

Natural killer (NK) cells are bone marrow-derived large granular cells that serve as critical effectors in innate immunity. During microbial challenge, activated NK cells mediate host defense through both cytolytic effector functions and immunoregulatory signaling (15). In sepsis, apoptosis of NK cells is accelerated, leading to a decrease in their number and cytotoxic function, in addition, excessive apoptosis of NK cells causes a decrease in the level of IFN-γ increasing the risk of secondary infection (16). And observational studies have found that peripheral blood PD-1+, PD-L1+NK cells can predict 28-day mortality in sepsis (17, 18). The role of NK cells in sepsis research has not yet been standardized and remains controversial: some studies have confirmed that NK cells have a protective role in sepsis, while others have suggested that NK cells are a risk factor in sepsis and exacerbate the process of sepsis (19). Addressing key knowledge gaps in NK cell biology throughout sepsis progression could unlock new opportunities for immunoadjuvant therapies tailored to the disease phase.

## 2 Inhibition of physiological functions and exhaustion of lymphocytes in sepsis

Among NK cells, B cells, and T cells, lymphocyte dysfunction can be broadly classified into several impaired states, including antigen-driven exhaustion, inadequate activation-associated anergy, and age-related senescence. These categories represent general immunological phenomena that describe how lymphocytes lose function under different physiological and pathological conditions (20). However, in the context of sepsis, accumulating evidence indicates that lymphocyte exhaustion and functional inhibition appear to represent central components of acute immune dysfunction in sepsis, rather than reflecting an equivalent contribution from all dysfunctional states. Persistent antigen exposure, overwhelming inflammatory signals, and profound metabolic stress collectively drive lymphocytes toward a progressive exhaustion phenotype, which plays a central role in sepsis-induced immunosuppression (21).

Lymphocyte exhaustion is characterized by a hierarchical loss of effector functions (IL-2 production, followed by TNF-α secretion and IFN-γ release), sustained overexpression of multiple inhibitory receptors (including PD-1, CTLA-4, TIM-3, and LAG-3), epigenetic reprogramming, and metabolic insufficiency caused by mitochondrial dysfunction. Importantly, exhaustion in sepsis represents a continuum rather than a terminal state, ranging from reversible functional impairment to irreversible cellular fate decisions (22). Within this continuum, exhaustion-associated apoptosis may represent an important mechanism linking functional suppression to the profound lymphopenia observed in septic patients. Although physiological apoptosis is essential for immune cell selection and homeostasis, persistent lymphocyte exhaustion in sepsis may predispose cells to apoptotic death through sustained cellular stress responses and cumulative replicative burden, thereby contributing to immune cell depletion during sepsis (23).

**Figure 2:**
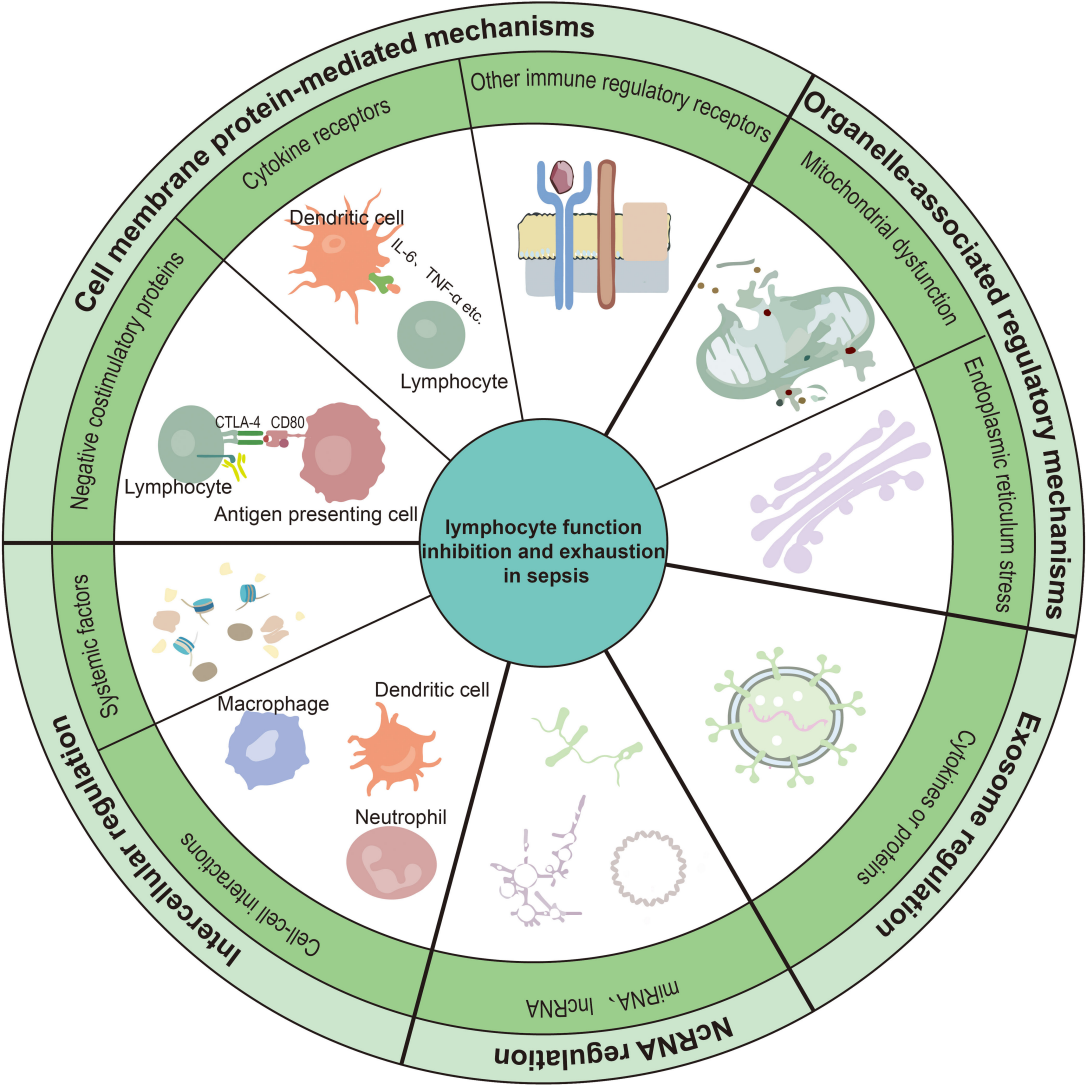
Mechanisms of lymphocyte function inhibition and exhaustion in sepsis. This schematic summarizes the major regulatory mechanisms contributing to lymphocyte function inhibition and exhaustion during sepsis. These include cell membrane protein-mediated mechanisms, encompassing negative costimulatory proteins, cytokine receptors, and other immune regulatory receptors; organelle-associated regulatory mechanisms, primarily involving mitochondrial dysfunction and endoplasmic reticulum stress; exosome-mediated intercellular regulation; and non-coding RNA-mediated regulatory mechanisms. In addition, other systemic and cell-cell interaction–mediated regulatory factors, such as circulating cell-free DNA, transcription factor dysregulation, and emerging metal ion-associated cell death pathways (including ferroptosis and cuproptosis), may further contribute to lymphocyte dysfunction in sepsis.

In addition to apoptosis, multiple forms of regulated cell death have been implicated in sepsis-associated immune dysfunction, including pyroptosis, ferroptosis, and cuproptosis. Pyroptosis is mediated by inflammatory caspases (such as caspase-1, -4, -5, and -11) and results in pro-inflammatory cell lysis, whereas ferroptosis is an iron-dependent form of cell death driven by dysregulated lipid peroxidation and redox imbalance (24–26). Cuproptosis, a recently described copper-dependent cell death pathway regulated by FDX1, disrupts mitochondrial protein homeostasis and cellular metabolism, further expanding the spectrum of exhaustion-associated immune cell loss (27). Emerging evidence suggests that sepsis-associated lymphopenia results not only from excessive lymphocyte death but also from combined defects in immune cell production within the bone marrow and thymus, as well as altered tissue distribution and migration. Together, these mechanisms converge to establish a state of sustained immunosuppression in sepsis (28). Based on this conceptual framework, the following sections focus on the molecular pathways and regulatory mechanisms that drive lymphocyte exhaustion and functional inhibition during sepsis (**Figure 2**).

## 3 Mechanisms of lymphocyte function inhibition and exhaustion in sepsis

### 3.1 Cell membrane protein-mediated mechanisms

#### 3.1.1 Negative costimulatory proteins (NCPs)

Immune checkpoints are cell-surface proteins that provide secondary signals to modulate antigen-specific immune responses, either enhancing or suppressing immunological activity. NCPs belong to an important class of proteins constituting the immune checkpoints, which transmit inhibitory signals after binding to their corresponding ligands to inhibit immune cell activation, proliferation, or functioning, thus negatively regulating the immune response (29).

##### 3.1.1.1 T cell immunoglobulin structural domain and mucin structural domain 3 (Tim-3)

The molecular structure of Tim-3 includes an IgV structural domain, a mucin-like structural domain, a transmembrane region, and an intracellular region containing tyrosine residues, and within the mucin structural domain, Tim-3 has only three predicted glycosylation sites (30). Tim-3, expressed on multiple immune cell populations including CD4+ T cells, CD8+ T cells, NK T cells, and monocytes, serves as a critical regulator of T cell homeostasis through modulation of activation thresholds, apoptotic pathways, and tolerance induction during sepsis (31). Tim-3 binds to its ligands in various immune responses and provides negative regulatory effects, inhibiting T cell immune responses and leading to T cell apoptosis, with the main ligands being phosphatidylserine (PtdSer), HMGB1, Galectin-9 (Gal-9), and Ceacam1 (32). During early sepsis, Tim-3 upregulation in NK T cells drives their hyperactivation and subsequent apoptosis. *In vitro* studies demonstrate that α-lactose-mediated blockade of the Tim-3/Galectin-9 interaction effectively rescues NK T cells from sepsis-induced apoptosis (33). Clinical analysis revealed a significant positive correlation between Tim-3+ CD4+ T cell frequency and mortality in sepsis-induced immunosuppression (*p* < 0.01). Mechanistically, the Tim-3/HMGB1 axis suppressed NF-κB signaling in Tim-3^+^ CD4^+^ T cells, potentially contributing to their dysfunctional state and suggesting a mechanistic basis for Tim-3–mediated regulation of sepsis-induced immunosuppression (34).

##### 3.1.1.2 B and T lymphocyte attenuator (BTLA)

Belonging to the immunoglobulin superfamily, BTLA contains a cytoplasmic immunoreceptor tyrosine-based inhibitory motif (ITIM) and shows predominant expression in B cells, T cells, dendritic cells, and monocytes (35). It negatively controls immune responses by engaging HVEM (herpesvirus entry mediator) and regulating multiple pathways (e.g., NF-κB, PI3K/AKT), thereby inhibiting cellular activation and proliferation (36). Pharmacological inhibition of PDGFR kinase was shown to upregulate BTLA expression in lymphocytes while suppressing CXCL13 and other chemokine secretion, ultimately leading to significantly improved survival outcomes in septic mice (37). The effects of BTLA expression vary at different stages of sepsis: at the pro-inflammatory stage, its expression increases with the enhancement of inflammatory response and may mitigate excessive inflammatory injury; at the anti-inflammatory stage, its high expression inhibits the activation of immune cells and over-expression of BTLA. During the anti-inflammatory phase, elevated expression levels were found to suppress immune cell activation. However, this excessive immunosuppression may precipitate secondary infections and worsen clinical outcomes. The precise mechanisms governing these immunoregulatory effects in sepsis remain to be fully characterized (38).

##### 3.1.1.3 Cytotoxic T lymphocyte antigen-4 (CTLA-4)

As an IgSF member, CTLA-4 contains the extracellular domain of the Ig-like structural domain as well as the cytoplasmic portion containing ITIM (immunoreceptor tyrosine-based inhibitory motif) and ITSM (immunoreceptor tyrosine-based switch motif), and is expressed predominantly on FoxP3 regulatory T cells, and activated T cells to inhibit T-cell activation. CD28, CTLA-4, and their shared ligands B7-1 (CD80) or B7-2 (CD86) form the CD28 pathway, and CTLA-4 mediates T-cell inhibition by competing for binding to the common ligand of the co-stimulatory receptor CD28 (39, 40). Studies reveal that mTOR-mediated dysregulation of autophagy-lysosome fusion represents a key mechanism governing CD4+ T cell apoptosis in septic conditions (41). Clinical investigations have identified a significant association between elevated CTLA-4 expression on CD4+ T cells and the development of sepsis-associated immunosuppression, and higher CTLA-4 levels have been independently associated with increased 28-day mortality (42). The role of CTLA-4 in the mechanism involved in lymphocyte function inhibition in sepsis is still unclear, and further studies are needed to reveal its specific regulatory mechanism and potential therapeutic value.

##### 3.1.1.4 Lymphocyte activation gene-3 (LAG-3).

LAG-3 also belongs to the immunoglobulin superfamily and consists of an extracellular region of four immunoglobulin-like structural domains, multiple potential phosphorylation sites, and an intracellular region of a conserved motif (43). LAG-3 is mainly expressed and up-regulated on B cells, T cells, activated NK and NK T cells, Treg cells, and DC cells (44), binding to the corresponding ligands MHC-II, Galectin-3, LSECtin, and FGL1 regulates T cell function and leads to their depletion (45). It has been demonstrated that LAG-3 downregulation or anti-LAG-3 antibody intervention attenuates sepsis-associated immune dysfunction in CLP mice, providing a basis for advancing clinically relevant experiments (46).

##### 3.1.1.5 Programmed death receptor-1 (PD-1)

PD-1 (CD279) is a protein with three main structural domains: a cytoplasmic region containing two tyrosine signaling motifs, an extracellular IgV-like domain, and a transmembrane domain. It acts as a critical inhibitor of both adaptive and innate immune responses and is primarily expressed on activated T cells, natural killer (NK) cells, B cells, macrophages, dendritic cells (DCs), and monocytes. PD-1 exerts its inhibitory effects by binding to its ligands, PD-L1 (CD274) and PD-L2 (CD273), which in turn suppress T cell activation, proliferation, and cytokine secretion (47, 48). Disruption of the PD-1/PD-L1 pathway suppresses lymphocyte apoptosis, thereby ameliorating sepsis-induced immunosuppression (49–51). Upregulation of the PD-1/PD-L1 axis during sepsis contributes to T-cell functional exhaustion by attenuating co-stimulatory signaling, thereby reducing T-cell responsiveness and promoting lymphopenia (52). Clinically, increased levels of soluble PD-1 and elevated PD-1 expression on memory CD8+ T cells have been associated with greater disease severity and higher 28-day mortality in septic patients (53, 54). In early sepsis, dysregulated PD-1/PD-L1 expression on antigen-presenting cells, including monocytes and dendritic cells, has been associated with organ dysfunction and mortality, with elevated PD-L1 and reduced PD-1 expression contributing to prognostic stratification (55). Collectively, these findings suggest that PD-1/PD-L1 signaling plays a central role in sepsis-associated immunosuppression and may serve as both a mechanistic mediator and a prognostic biomarker.

##### 3.1.1.6 T cell immunoglobulin and ITIM domain (TIGIT)

TIGIT is composed of an extracellular immunoglobulin variable domain, a type I transmembrane domain, and a short intracellular domain containing an immunoreceptor tyrosine-based inhibitory motif (ITIM). It is predominantly expressed on T cells, natural killer (NK) cells, and regulatory T cells (Tregs). The ITIM domain mediates inhibitory signaling through recruitment of phosphatases (e.g., SHP-1/2) upon phosphorylation. This leads to dephosphorylation of key signaling molecules in the TCR/CD28 pathway, ultimately suppressing T cell activation and cytokine production. Upon binding to ligands such as CD155, CD112, and CD114 (Nectin4), TIGIT inhibits the activity of T cells and NK cells via this ITIM-dependent mechanism. Additionally, it enhances the function of regulatory T cells through various signaling pathways, including NF-κB, MAPK, and PI3K/AKT, where the ITIM collaborates with other intracellular motifs to amplify immunosuppressive signals (56–58).

TIGIT+ Tregs continued to expand in advanced sepsis, and their expansion was dependent on the IL-33/ST2/STAT6/M2 macrophage axis and correlated with the immunosuppressive state (59). TIGIT expression was significantly upregulated on T cells in sepsis and sepsis-recovered patients, correlating with increased inflammatory response and organ damage, and TIGIT blockade restored T cell function, suggesting its potential as a prognostic marker and immunotherapeutic target (60). TIGIT expression is also increased during polymicrobial sepsis, where it correlates with CD4+ T-cell dysfunction and may compromise resistance to secondary infections, thereby adversely affecting clinical prognosis (61).

However, emerging evidence indicates that therapeutic targeting of the TIGIT pathway exhibits fundamental differences depending on the immune status of the host. In experimental models, αTIGIT antibody treatment had distinct effects in “naïve mice” with no prior immunological experience versus “memory mice” with previous immunization or sepsis exposure. While αTIGIT antibody did not improve survival in septic mice without prior sepsis, it significantly worsened outcomes in mice with a history of sepsis (62). These findings suggest that the role of TIGIT in sepsis is highly context-dependent, and that immunotherapeutic strategies targeting TIGIT may require careful stratification based on host immune memory and prior exposure history.

##### 3.1.1.7 V-type immunoglobulin domain-containing suppressor of T cell activation (VISTA)

VISTA is consists of an extracellular domain rich in histidine residues, a transmembrane domain, and an intracellular domain that is predominantly expressed on CD4+, CD8+ T cells, NK cells, dendritic cells, and it is mainly localized on CD4+, CD8+ T cells, NK cells, dendritic cells, and macrophages, exists in both transmembrane and soluble forms, and restricts T cell stimulation and enhances the inhibitory activity of Tregs when bound to ligands such as VSIG-3 and Galectin-9. These pathways, such as PI3K/AKT and MAPK, play a role in the process. As a receptor: Binds canonical ligands VSIG-3 and Galectin-9 to inhibit T cell activation via PI3K/AKT and MAPK pathways. As a ligand: Engages PSGL-1 on myeloid cells to suppress inflammatory responses (63, 64).

In experimental sepsis models, binding of the high-affinity anti-VISTA antibody MH5A to the VISTA receptor attenuated CD3+ T-cell apoptosis and inhibited lipopolysaccharide (LPS)-induced macrophage activation in cecal ligation and puncture (CLP) mice (65). Conversely, Gray et al. reported that enhanced VISTA expression or adoptive transfer of VISTA+ Tregs during early sepsis mitigated inflammatory mortality, suggesting a protective role of VISTA-mediated immune inhibition in the hyperinflammatory phase of sepsis (66).

These findings indicate that the immunological impact of VISTA varies across the course of sepsis. During the early hyperinflammatory stage, VISTA may protect tissues and organs by limiting excessive immune activation and antigen presentation. In contrast, during the late immunosuppressive phase, persistent VISTA-mediated inhibitory signaling may exacerbate immune paralysis, impair pathogen clearance, and hinder tissue repair. This stage-dependent duality highlights the complexity of targeting VISTA in sepsis therapy and underscores the need for temporal stratification in immunomodulatory interventions.

Recent studies highlight VISTA’s intracellular roles beyond its surface expression as an immune checkpoint regulator. Stored in vesicles and aligned with microtubules, VISTA rapidly translocates to the cell surface upon immune stimuli, similar to CTLA-4. It is also secreted in exosomes, potentially suppressing T-cell activity in the tumor microenvironment (67). Nuclear localization of VISTA suggests possible transcriptional or anti-apoptotic roles. Intracellularly, VISTA interacts with Galectin-9 and TAK1 to protect lysosomal integrity and regulate autophagy, with transforming growth factor-β (TGF-β) modulating these interactions in a cell type–dependent manner (68). These features suggest that VISTA surface expression is transient and context-dependent, which may influence responsiveness to immune checkpoint blockade. Therefore, effective targeting of VISTA should consider its intracellular reservoir and exosomal secretion, as these mechanisms may contribute to immune checkpoint inhibitor resistance.

##### 3.1.1.8 Natural killer cell receptor 2B4 (CD244/SLAMF4)

**Figure 3:**
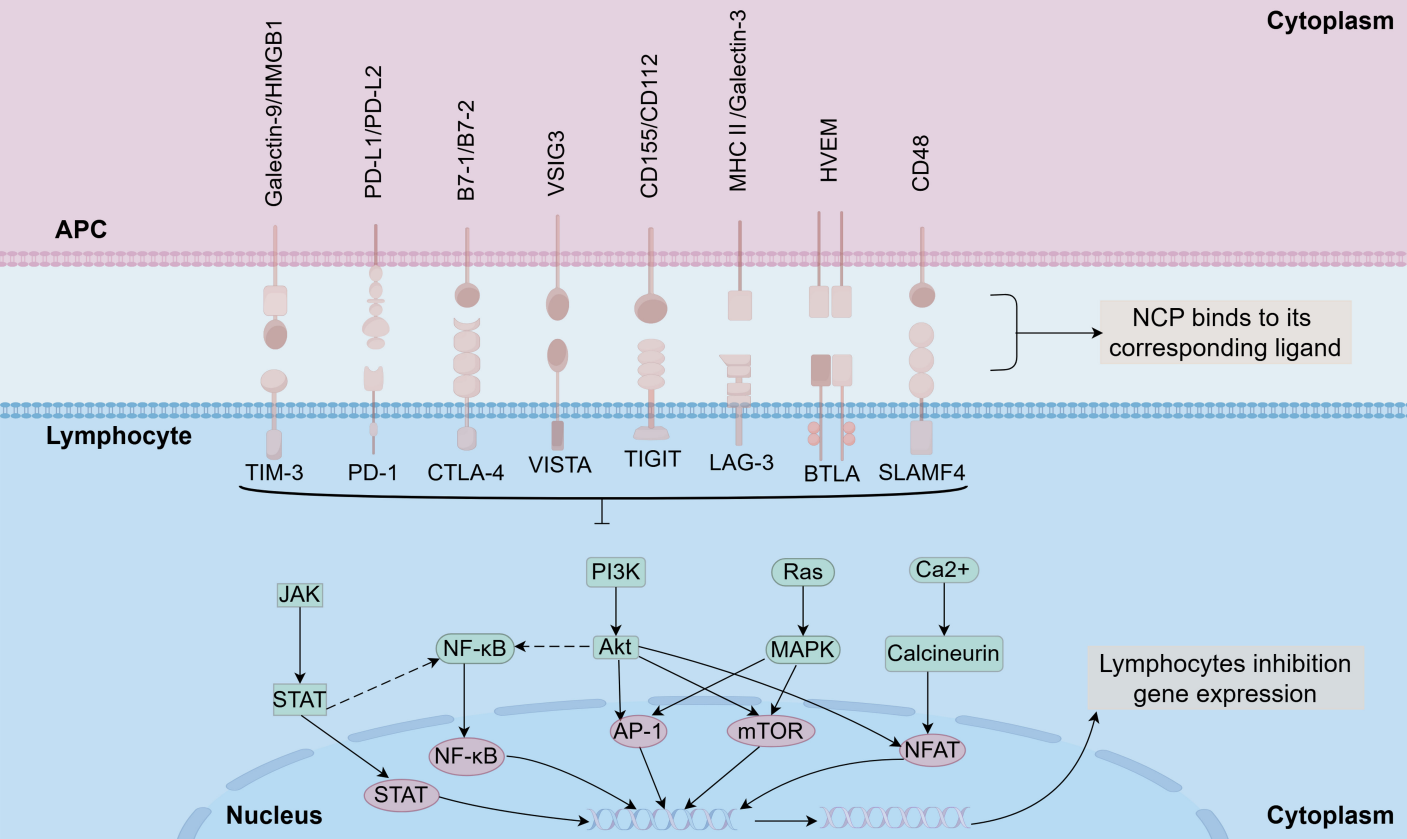
Molecular mechanistic pathways associated with immune checkpoints in lymphocytes.

2B4 is a receptor belonging to the signaling lymphocyte activation molecule (SLAM) family. It comprises an extracellular Ig-like domain, a transmembrane region, and a cytoplasmic domain, and is primarily expressed on natural killer (NK) cells and T cells. CD48 is a well-established ligand for 2B4. Elevated expression levels of these molecules may lead to sepsis-induced immunosuppression by impairing immune cell function and promoting a state of immune suppression. Interrupting the interaction between 2B4 and its ligand could potentially help replenish T cells depleted by sepsis (69–71). 2B4 expression promotes apoptosis of memory CD4+ T cells during sepsis, and targeting 2B4 co-inhibitory signaling may be a potential immune-modulating therapy (72). **Figure 3** provides an overview of the mechanisms through which these negative costimulatory proteins (NCPs) mediate lymphocyte dysfunction and contribute to sepsis-induced immunosuppression (**Figure 3**).

**Figure 4:**
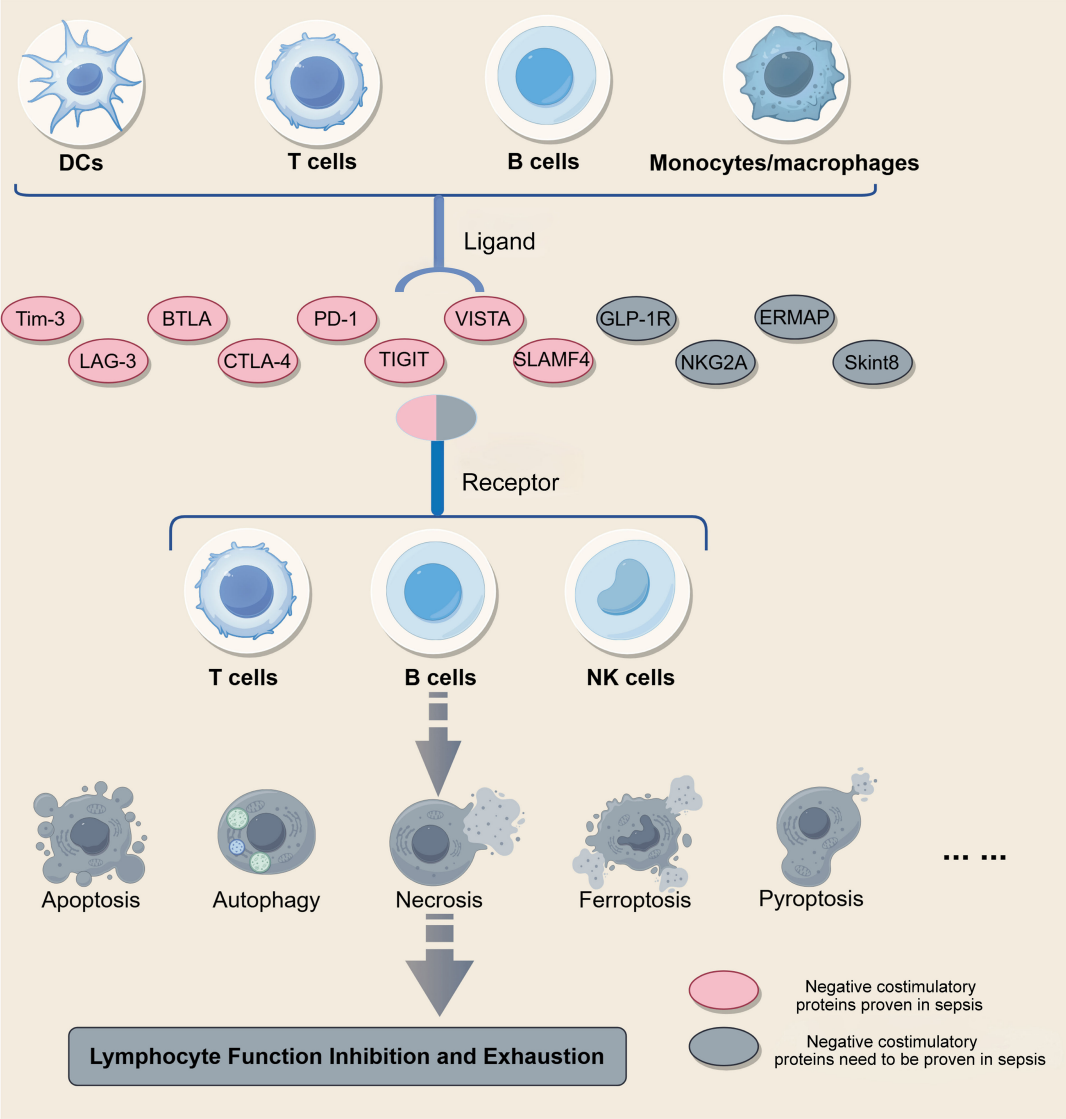
Immune checkpoint networks between antigen-presenting cells and lymphocytes. This schematic delineates the ligand-receptor interactions involving negative costimulatory proteins in immune regulation. Receptors such as Tim-3, LAG-3, BTLA, CTLA-4, PD-1, TIGIT, VISTA, and SLAMF-4 are expressed on T cells, B cells, and NK cells, while their corresponding ligands are presented by dendritic cells, monocytes/macrophages, and other immune cells. The engagement of these ligand-receptor pairs suppresses lymphocyte activation and may directly or indirectly contribute to cell death pathways such as apoptosis, autophagy, necrosis, ferroptosis, and pyroptosis. While some negative costimulatory proteins have been implicated in sepsis-induced immune dysfunction, the roles of others remain to be validated, and further studies are needed to elucidate their mechanistic links to specific cell death processes.

Numerous studies have demonstrated that sepsis and tumor-induced immunosuppression share fundamental mechanistic principles, particularly in sustained inhibitory receptor signaling, metabolic reprogramming, and progressive lymphocyte dysfunction. Although the initiating triggers and temporal dynamics differ between these conditions, convergent immune regulatory programs have been documented at the cellular and molecular levels (10, 73). In this regard, several inhibitory receptors and regulatory molecules that have been extensively studied in oncology, such as NKG2A (74), metabolic modulators including GLP-1R (75), and B7 family-related members such as ERMAP (76) and Skint8 (77), have demonstrated the capacity to restrain T-cell activation or reshape immune metabolism in other disease settings. While direct evidence linking these molecules to sepsis remains limited, their established roles in modulating immune inhibitory networks suggest that they warrant further investigation in the context of sepsis-associated immune dysfunction. Systematic investigation of these shared immune regulatory networks may not only improve the understanding of lymphocyte dysfunction in sepsis but also facilitate the rational repurposing or refinement of immunomodulatory strategies for septic patients (**Figure 4**).

#### 3.1.2 Cytokine receptors

Among the pro-inflammatory factors, TNF acts as a core cytokine of the inflammatory response (e.g., the classical pro-inflammatory factors TNF-α, TNF-β) and binds to its TNF receptor 1 (TNFR1) and TNFR2 to induce cell death (including apoptosis, necrosis, and pyroptosis). TNF-induced cell death releases DAMPs (damage-associated molecular patterns), activates PRRs (pattern-recognition receptors), triggers the NF-κB and MAPK signaling pathways, increasing inflammatory gene expression and exacerbating the pathological process of sepsis, which may lead to organ damage and failure (78). TIPE1, a novel member of the tumor necrosis factor-α-induced protein 8 family, may exert negative regulation in sepsis. This effect is partly achieved through blocking dendritic cell (DC) maturation and ensuing T-cell-mediated immunity via PD-L1/PD-1 signaling (79). Kim et al. reported that although IFN-γ classically exerts pro-inflammatory and antimicrobial effects, elevated endogenous IFN-γ production during early sepsis was associated with impaired macrophage phagocytosis and increased susceptibility to secondary infection via an NKT cell–mTOR regulatory axis, suggesting that dysregulated IFN-γ signaling may contribute to post-sepsis immune dysfunction (80). In addition, synergistic signaling between TNF-α and IFN-γ has been shown to induce inflammatory cell death characterized as PANoptosis, resulting in tissue injury and mortality. Mechanistically, co-stimulation with TNF-α and IFN-γ activates the JAK/STAT1/IRF1 signaling axis, promotes nitric oxide production, and drives caspase-8/FADD-mediated PANoptosis. In murine models, this cytokine synergy induces lethal cytokine shock resembling severe inflammatory pathology. Importantly, combined neutralization of TNF-α and IFN-γ with specific antibodies significantly reduced mortality during SARS-CoV-2 infection, sepsis, hemophagocytic lymphohistiocytosis, and cytokine shock syndromes (81).

It is worth noting that interleukin-6 (IL-6) no longer functions solely as a classical pro-inflammatory cytokine but rather as a pleiotropic mediator whose effects depend on the immune context. Upon binding to membrane-bound IL-6 receptor, IL-6 activates intracellular JAK/STAT and Ras/MAPK signaling pathways, which may disrupt lymphocyte survival programs and increase apoptosis under septic conditions (82). Among the anti-inflammatory factors, IL-4 is involved in signaling via cell surface receptor complexes and can inhibit pro-inflammatory cytokine formation such as TNF and IL-6, which both suppress acute inflammation and induce persistent innate immune memory. IL-4 nanotherapy developed by Schrijver’s team can address immune paralysis due to lipopolysaccharide-induced hyperinflammation in mice or may be beneficial for septic patients (83). IL-10 as a classical antigenic factor in combination with its receptor both regulates the local cytokine microenvironment and limits antigen presentation, downregulating T cells activity, however, various studies have confirmed that IL-10 is a cytokine with pleiotropic effects, exerting immunostimulatory activity, increasing B-cell survival, and promoting B-cell proliferative activation depending on the degree of inflammation and the concentration of IL-10, and the kind of target cells, stage of immune response (84, 85). Transforming growth factor-β (TGF-β) is also included, regulating T cell differentiation, inhibiting effector T cells, and promoting the formation of memory T cells, which helps to maintain the balance and stability of the immune system (86). Notably, the biological effects of IFN-γ in sepsis appear to be highly context-dependent. In contrast to endogenous overactivation, controlled exogenous administration of IFN-γ in experimental models has been shown to partially restore immune competence, reduce T-cell apoptosis, and modulate regulatory T-cell expansion (87), indicating that the timing, dose, and immune phase critically determine whether IFN-γ exerts detrimental or protective effects.

#### 3.1.3 Other immune regulatory receptors

Calcium-sensitive receptprotects mice from novel coronavirus infection, hemophagocytic lymphohistiocytosis (HLH), and sepsis aggressionor (CaSR), which belongs to the G protein-coupled receptor superfamily, is present in lymphocytes and promotes cytokine secretion. In sepsis, CaSR may induce T-cell apoptosis and the secretion of both pro-inflammatory cytokine TNF-α and anti-inflammatory cytokine IL-4 via the NF-κB pathway and components of the MAPK signaling pathway (88). CXCR4 is a chemokine receptor, and inhibition of CXCR4 mitigates CD4+ T cell loss during sepsis and enhances survival in a mouse model of polymicrobial sepsis (89). Epidermal growth factor receptor (EGFR) regulates Glut1 translocation to the cell surface through the TBK1/Exo84/RalA protein system, inducing the Warburg effect and promoting CD4+ T cell activation and apoptosis, which may exacerbate immune cell depletion in sepsis (90). β1-adrenergic receptor activation amplifies the inhibitory function of regulatory T cells (Treg cells), leading to sepsis-induced immunosuppression, and β1-adrenergic receptor blockade reverses this effect, suggesting a potential immunomodulatory role in sepsis treatment (91). Tumor necrosis factor receptor 2-positive (TNFR2+) Treg subpopulations support most suppressive effector functions, and inhibition or removal of TNFR2 during sepsis reduces the susceptibility to secondary infections, which could recognize TNFR2-positive Treg as a target for therapeutic intervention (92). P2Y (12) is a nucleotide receptor expressed on platelets and T cells. Inhibition of the P2Y (12) signaling cascade suppresses Treg cell proliferation both *in vivo* and *in vitro*. Aiming at platelets as a promising approach to regulate Treg cell proliferation and activity may be beneficial in the treatment of sepsis (93).

Collectively, despite structural differences, negative costimulatory receptors converge on shared downstream pathways. Sustained activation of PD-1, BTLA, LAG-3, and related molecules suppresses PI3K/AKT/mTOR signaling, dampens NF-κB activity, and disrupts mitochondrial metabolism, leading to reduced cytokine production, impaired proliferation, and functional exhaustion of lymphocytes in sepsis. This convergence links inhibitory receptor overexpression to the progressive immune dysfunction observed in septic patients (94–96).

### 3.2 Organelle-associated regulatory mechanisms

#### 3.2.1 Mitochondrial dysfunction

Mitochondria are crucial cellular components for energy generation in cells and also serve a critical function in the control of programmed cell death (97). Lymphocytes from sepsis patients suffer from increased mitochondrial membrane permeability and oxidative stress, which can impair the mitochondrial respiratory chain and mtDNA, which subsequently induces mitochondrial damage and dysfunction, ultimately contributing to apoptosis in lymphocytes. Impaired function of mitochondrial components, including protein content, mtDNA concentration, oxidative complex activity, and F1Fo-ATP synthase, is associated with this condition (96, 98). Given these features, strategies such as mitochondria-targeted drugs, mitochondrial autophagy stimulators, modulators of mitochondrial kinetics, and mitochondrial biogenesis enhancers may have therapeutic potential. The promising biological marker function of mitochondria in sepsis prognosis and recovery, as well as its association with different organ failures, also deserves more exploration and evidence in the future.

#### 3.2.2 Endoplasmic reticulum stress

Endoplasmic reticulum stress is an adaptive response of cells to endoplasmic reticulum dysfunction, and excessive endoplasmic reticulum stress can lead to apoptosis (99). The evolutionarily conserved protein kinase mTOR serves as a key regulator of apoptosis. The results showed that apoptosis of CD4+ T cells is induced by endoplasmic reticulum stress through the mTOR-Akt-IRE1α-JNK signaling pathway in septic mice (95). STING is a protein-coding gene located in the endoplasmic reticulum membrane, and it was found that calcium signaling may be a synergistic point of action for STING gain-of-function mutation-mediated T cell death, and endoplasmic reticulum stress may be a significant mediator of downstream T cell death (100). Inhibition of endoplasmic reticulum stress reduces CD4+ T cell apoptosis and maintains CD4+ T cell function in sepsis, and may ameliorate immunosuppression in advanced sepsis (101).

Ribosomal autophagy selectively degrades dysfunctional or excess ribosomes to maintain cellular homeostasis. In sepsis, NUFIP1-mediated ribosomal autophagy is significantly activated via the PERK-ATF4-CHOP pathway, thereby reducing T cell apoptosis. Thus, targeting NUFIP1-mediated ribophagy may be crucial for reversing sepsis-induced immunosuppression in critical illnesses (102).

### 3.3 Exosome-mediated intercellular regulation

Exosomes are extracellular vesicles with a diameter of 30–200 nm that are released by diverse cell types and contributing significantly to intercellular communication and a variety of physiopathological processes because they contain proteins, lipids, glycoproteins, non-coding RNAs, and DNA, etc. (103). The study analyzed the miRNA and mRNA content of exosomes in the plasma of septic shock patients, and found that the molecules carried by these exosomes are related to pathological pathways such as inflammatory reaction, oxidative strain and cell cycle control, suggesting that exosomes may act as a novel mechanism for cell-to-cell communication during sepsis (104). Extracellular vesicles (EVs) have dual roles in inflammation, lymphocyte apoptosis, coagulation and organ dysfunction during sepsis pathophysiology (105). An animal study found that fresh frozen plasma (FFP) infusion remodeled the immune response of CD4+ T cells in severely septic mice by inhibiting the secretion of the exosomal protein Galectin-9, promoting the proliferation of Th1 and Th17 cells, suppressing Treg cells, and modulating the secretion of inflammatory factors, thereby alleviating the immunosuppressive state (106). Therefore, it has been proposed that exosomes secreted by sepsis-associated immune cells or non-immune cells may carry pro-inhibitory factors (e.g., pro-inflammatory cytokines or miRNAs), thereby promoting lymphocyte function inhibition (107). Conversely, extracellular vesicles may also exert protective effects in sepsis through cargo-dependent immune modulation. Protein cargos derived from macrophage EVs have been shown to regulate inflammatory signaling, and ASC-containing pyroptotic EVs can preserve B-cell viability in septic models, collectively suggesting a potential role for EVs in restoring lymphocyte function (108, 109). Exosomes derived from human umbilical cord mesenchymal stem cells (HUCMSCs-Exos) were shown to decrease CD4+ T cell apoptosis and enhance autophagy by transferring miR-375, which inhibits the expression of HDAC4 (HDAC4, a zinc-dependent class II histone deacetylase, is a significant regulator of gene expression) (110). Extracellular vesicles (pyroEVs) generated by pyroptosis cells have a unique pyroptosis marker ASC expression, which can protect the survival of B cells by inhibiting Toll-like receptor 4, decrease the inflammatory reaction and enhance the survival rate of septic mice (109). A recent study examined serum exosomal components in patients with sepsis and septic shock and discovered that miR-193b-5p has the potential to serve as a diagnostic indicator for sepsis and is linked to inflammation and immunomodulation in sepsis (111).

### 3.4 Non-coding RNA-mediated regulatory mechanisms

Recent studies have shown that several types of RNAs, such as miRNAs and lncRNAs, play a significant role in regulating the immunosuppressive state of lymphocytes in sepsis. Elevated miR-155 expression in sepsis patients has been shown to correlate positively with SOFA scores, disease severity, reduced 28-day survival, and the proportion of CD39(+) Tregs. Experimental inhibition of miR-155 in septic mice decreased CD39(+) Treg frequency, supporting a potential role for miR-155 in promoting sepsis-associated immunosuppression through expansion of regulatory T cells (112). miR-31 affects T cell inflammatory phenotype and cytokine secretion by modulating NF-κB, FIH-1α and SLAM receptor signaling pathways, and its down-regulation in septic patients is connected to immunosuppression (113). Clinical studies have also suggested that miR-143 and miR-150 could be potential indicators for detecting T cell immunosuppression in peripheral blood (114). In septic rats, overexpression of miR-126 was associated with reduced pro-inflammatory cytokine release, a shift toward Treg differentiation with concomitant suppression of Th17 polarization, and decreased caspase-dependent lymphocyte apoptosis. In contrast, inhibition of miR-126 produced the opposite effects, supporting a role for miR-126 in regulating Th17/Treg balance and lymphocyte viability during sepsis (115). Serum miR-150 expression was found to be reduced in both sepsis patients and mice. Supplementation with miR-150 was shown to inhibit the expansion of MDSCs (myeloid-derived suppressor cells) in both monocytes and polymorphonuclear subpopulations. Additionally, it reduced the immunosuppressive function of MDSCs by downregulating ARG1 (116). miR-223 can protect against sepsis by reducing lymphocyte apoptosis and promoting lymphocyte proliferation through its interaction with FOXO1 (117). In septic mice, lncRNA NEAT1 upregulates MCEMP1 expression by inhibiting miR-125, which suppresses T cells activity and NK cells activity and promotes the release of inflammatory factors and T cell apoptosis (118). The late sepsis lncRNA HOTAIRM1 is up-regulated through the Notch/Hes1 signaling pathway and regulates PD-L1 expression through HOXA1, inducing T cell depletion and the development of an immunosuppressive microenvironment, and blockade of this axis alleviates lung injury and improves survival in mice (119). These non-coding RNAs may alter sepsis-associated immunosuppression by affecting processes such as lymphocyte activity, inflammatory factor release, and apoptosis, providing new potential targets for the treatment of sepsis.

### 3.5 Cell-cell interaction-mediated and systemic regulatory mechanisms

Myeloid-derived suppressor cells (MDSCs) represent a heterogeneous group of immature myeloid cells (IMC) with significant immunosuppressive functions. Sepsis-induced immunosuppression begins at an early stage with MDSCs that highly express PD-L1, and their major subunit PMN-MDSC may inhibit T-cell proliferation and promote apoptosis through the PD-L1/PD-1 axis (120). If the expansion of MDSC and its tissue infiltration persist, it may cause essential pathophysiologic changes, involving lymphopenia, host immunosuppression, and immunoparalysis, leading to a deterioration of patient prognosis (121).

Neutrophils and T cells are significantly connected to the development of immunosuppression in sepsis. CD10 is a reliable biomarker for distinguishing between mature and immature neutrophils in sepsis patients. Mature CD10+ neutrophils inhibit T-cell proliferation and immature CD10^−^neutrophils promote T-cell proliferation (122). It has been confirmed that pro-inflammatory factors and the high mobility histone HMGB1, which is passively released by dead cells, upregulate PD-L1 via toll-like receptor 2 (TLR2) on neutrophils, leading to boosted apoptosis of T cells, immune dysfunction, and ultimately the development of immune function inhibition in sepsis (123).

In a CLP-induced murine model of sepsis, VSIG4-expressing peritoneal macrophages migrate actively toward the thymus and trigger apoptosis of double-positive thymocytes, resulting in thymic involution. Regulation of VSIG4-expressing macrophages may be an effective strategy to prevent and manage immune suppression induced by polymicrobial infection (124). An IgG-like receptor called signaling lymphocyte activation molecule family 7 (SLAMF7) was found to be a critical inhibitor of inflammation during sepsis, and it was demonstrated that SLAMF7 prevents lethal sepsis through down-regulation of macrophage pro-inflammatory cytokines and inhibition of inflammation-induced organ damage (125).

Overexpression of platelet MHC-I during sepsis was found to increase antigen cross-presentation and interaction with CD8+ T cells in an antigen-specific manner. In sepsis, platelets bind to antigen-specific CD8+ T cells through MHC-I processing and cross-presentation of antigen. Through this mechanism, the upregulation of MHC-I on platelets during sepsis leads to the suppression of CD8+ T cell numbers, proliferation, and functional responses (126). At the same time, the functional status of lymphocytes may also influence the activity of these regulatory cells, creating an immunomodulatory feedback, which requires further research investigations to reveal the complex pathophysiologic processes involved in sepsis.

The selection and apoptosis of CD4+CD8+ double-positive thymocytes are significantly regulated by glucocorticoids (GC). GC-induced accumulation of mitochondrial Bax and the interaction of glucocorticoid receptor (GR) with Bim, Bcl-xL, and Bak may be engaged in the regulation of thymocyte apoptosis (127). Cell-free DNA is a circulating extracellular DNA fragment induced by cell necrosis, apoptosis, pyroptosis and NETosis, a program that forms extracellular traps in neutrophils (128). The study comprehensively analyzed the amount of free DNA (cfDNA), fragmentation patterns, pathogen detection and microbial composition of plasma from sepsis patients by macro-genome sequencing, and combined it with a machine learning model to enable early diagnosis and prediction of clinical prognosis in sepsis (129). It was demonstrated that down-regulation of the expression of the transcription factors T cell factor 7 (TCF7) and lymphoid enhancer factor 1 (LEF-1) in sepsis inhibits CD4+ T cell proliferation and leads to immunosuppression. This observation suggests that TCF7 and LEF-1 are promising therapeutic targets for ameliorating lymphocyte function inhibition in sepsis, and suggests that immunotherapy targeted at improving CD4+ T-cell proliferation may be a novel approach for immunotherapy in septic patients (130). Sepsis activates the caspase cascade reaction, and activation of caspase-3, a key protease in the caspase cascade reaction, can lead to the development of apoptosis. Lymphocyte caspase-3 activity is increased in sepsis patients, and inhibition of caspase-3 activity reduces lymphocyte apoptosis, further suggesting that caspase-3/GSDME could be a viable target for sepsis research (131). Protein arginine methyltransferase (PRMT4) is elevated in B and T cells as well as THP-1 monocytes from sepsis patients, and aberrant expression of PRMT4 protein leads to massive lymphocyte apoptosis through caspase-3 signaling, which may provide clues for designing therapeutic strategies to attenuate sepsis-induced immunosuppression (132).

Taken together, negative costimulatory signaling in sepsis should not be interpreted as isolated checkpoint events but rather as components of a coordinated immunosuppressive network. Persistent upregulation of inhibitory receptors integrates with mitochondrial dysfunction, metabolic reprogramming, and epigenetic modulation, forming a multilayered regulatory system that governs lymphocyte fate. Non-coding RNAs and extracellular vesicle-mediated signaling may further stabilize or amplify these inhibitory circuits, contributing to sustained immune paralysis. This network-based perspective helps reconcile molecular findings with clinical observations of lymphopenia, impaired effector responses, and increased susceptibility to secondary infections in sepsis (97, 133, 134).

## 4 Application of lymphocytes in early diagnosis, immune function assessment, and prognosis evaluation of sepsis

It has been found that sepsis is the main cause of lymphopenia in hospitalized patients and that lymphopenia is strongly connected with increased susceptibility to infection, mortality from infection, and prognosis of sepsis, and that detection of lymphocyte function inhibition levels may assist in risk stratification, evaluating its severity, and guiding clinical treatment. Lymphocyte count plays an important role as a potential, affordable, fast, and easily accessible biomarker for the diagnosis of sepsis. Sepsis-associated lymphopenia is typically characterized by an absolute lymphocyte count in the peripheral blood of < 1,000 cells/uL. When a nonviral infection is suspected and the level of lymphopenia is below the optimal threshold value of 760 cells/uL, a high degree of vigilance is required for the presence of sepsis (135–137).

Persistent severe lymphopenia (< 760 cells/μL) lasting for more than three days has been shown to predict a significantly increased 28-day mortality in septic patients (138). Earlier clinical studies also found that hypothermia within 24 hours of sepsis diagnosis was related to higher mortality and increased risk of persistent lymphopenia, and may serve as an early clinical predictor of sepsis-induced immunosuppression (139). Conversely, the functional mechanism of hypothermia and lymphocytopenia remains to be discovered by further studies. Several early screening tools for sepsis have been developed, including the Sequential Organ Failure Assessment (SOFA), quick SOFA (qSOFA), Modified Early Warning Score (MEWS), and National Early Warning Score (NEWS) (140). In a previous single-center study, our group proposed a novel screening approach based on lymphocyte count, International Normalized Ratio (INR), and procalcitonin levels, termed the LIP score. This model demonstrated potential utility for rapid sepsis screening in emergency outpatient settings and resource-limited regions (141).

Apart from the absolute lymphocyte count, innate lymphocyte subpopulations reflect the initial activation of intrinsic immunity, effector T cells reflect the attacking capacity of cellular immunity, B cells demonstrate the potential for antibody production in humoral immunity, and dendritic cells are an significant measure of antigen presentation and immune initiation, which, taken together, make possible the assessment of the state of immune function from a variety of perspectives (28, 142, 143). The logistic regression analysis revealed that low CD8+ T-cell counts are associated with an increased risk of sepsis progression (144).

Compared with more complex immunophenotyping approaches such as CD8+ T-cell subset analysis, simpler indices including the neutrophil-to-lymphocyte ratio (NLR) are widely accessible and have demonstrated prognostic value for short-term mortality in sepsis (145). However, while NLR reflects systemic inflammatory imbalance, it does not capture functional exhaustion or phenotypic alterations of specific lymphocyte subsets. In addition, neutrophil counts do not increase in a strictly linear manner with infection severity, and substantial variability in neutrophil expansion and lymphocyte decline has been observed across adult sepsis cohorts. Consequently, reported optimal NLR cut-off values for predicting severity or mortality vary considerably between studies, ranging from moderate thresholds for sepsis identification to higher values for prognostic stratification, with only modest and inconsistent sensitivity and specificity overall (146, 147). A large meta-analysis of adult sepsis patients further confirmed that elevated NLR is associated with poor prognosis but highlighted heterogeneity across studies in terms of designs and cut-off values, which may limit reproducibility and complicate standardization in clinical practice (148). In contrast, reductions in CD4+ and CD8+ T-cell counts have been associated with adverse outcomes (149), suggesting that lymphocyte subpopulation analysis may provide additional mechanistic insights, although its incremental predictive value over conventional indices requires further validation.

The septic lymphocyte apoptosis-associated negative costimulatory proteins LAG3 and PD-1 have a potential cooperative function in modulating the progressive T-cell exhaustion, which severely affects the clinical prognosis of patients. T cells showing double positivity for LAG3 and PD-1 are important indicators for immune testing and prognostic evaluation (150). Beyond cell subset quantification, additional composite hematological indices, including LMR, have also demonstrated prognostic relevance (151). A single-center observational study reported that the frequency of CD4+ T cells expressing intracellular mTOR and IFN-γ, as well as surface PD-1, was associated with 28-day mortality in septic ICU patients; however, these findings require further validation in larger cohorts (152). The epigenetic regulator histone methyltransferase EZH2 was markedly elevated in CD4+ and CD8+ lymphocytes in sepsis patients. Increased EZH2 expression was associated with higher mortality and secondary infectious complications, suggesting its potential as a prognostic biomarker (153). Hypoxia-inducible factor-1α (HIF-1α) induces upregulation of sialic acid-binding immunoglobulin-like lectin 5 (SIGLEC5) in monocytes, and SIGLEC5–PSGL1 interactions inhibit CD8+ T-cell proliferation. Circulating sSIGLEC5 levels were associated with clinical outcomes, and blockade of the SIGLEC5/PSGL1 axis has been proposed as a potential immunomodulatory strategy in sepsis (154).

These mechanistic insights into lymphocyte function inhibition and exhaustion in sepsis have unveiled a diverse array of potential therapeutic targets and pharmacological agents (**Table 1**). Notably, in addition to traditional chemically synthesized drugs, more and more studies have revealed the unique value of traditional Chinese medicines and natural extracts in the treatment of sepsis. For example, genipin (the main active ingredient in the fruit of Gardenia jasminoides) and HS-23 (a honeysuckle extract) attenuate sepsis-induced immunosuppression by suppressing apoptosis of lymphocytes in mice (155, 156). Rhodiola rosea enhances immunity and improves survival of septic mice by down-regulating TIPE2 expression, inhibiting apoptotic genes, decreasing T cell apoptosis, and increasing Th1 cytokine levels (157). Hydroxy saffron yellowness A (HSYA) attenuates sepsis-induced apoptosis of CD4+ T cells and ameliorates immunosuppression via anti-inflammatory and anti-apoptotic effects (158). These preclinical findings suggest potential immunomodulatory effects; however, clinical translation remains uncertain, as most evidence derives from small animal models and high-quality randomized clinical trials are lacking, due to limitations in bioavailability, dosing standardization, and safety evaluation. Nevertheless, these findings lay the foundation for further exploration of the mechanisms of action of natural compounds and their clinical applications.

**Table 1:** Sepsis lymphocyte function inhibition therapeutic targets and applications summary.

Target name	Mechanistic category	Cell type	Function	Research stage	Survival rate	Model	Intervention	Reference
IL-7	Cytokine signaling	T cells	Promotes lymphocyte proliferation and prevents lymphocyte apoptosis	Clinical trial	–	Human sepsis cohort	CYT107	(159)
PD-L1	Immune checkpoint	T cells	Prevents T cells death and regulates cytokine production	Clinical trial	–	Human sepsis cohort	BMS-936559	(160)
PD-1	Immune checkpoint	T cells	Enhances immune response, inhibits T cells exhaustion	Clinical trial	–	Human sepsis cohort	Nivolumab	(161)
IL-38	Cytokine signaling	CD4+ CD25+ Tregs	Enhances the immunosuppressive activity of CD4+CD25+ Tregs	Preclinical animal model	↑	Murine CLP model	rmIL-38	(162)
Tim-3	Immune checkpoint	NKT cells	Inhibits NK T cell apoptosis *in vitro* in septic patients	Translational study (human + murine model)	–	Human sepsis cohort and murine CLP model	α-lactose	(33)
PDGFR	Receptor signaling	T cells、B cells	Promotes the expression of BTLA and suppresses the release of chemokines including CXCL13	Preclinical animal study	↑	Murine CLP model	CP-673451	(37)
mTOR	Metabolic regulator	CD4+T cells	Alleviates CD4+ T-cell dysfunction by reducing CTLA4 accumulation and rescuing autophagy	Preclinical animal study	–	Murine CLP model	Rapamycin	(163)
LAG-3	Immune checkpoint	T cells	Enhances the secretion ability of IFN-γ in CD4+ T cells as well as the proliferation of CD4+ and CD8+ T cells	Preclinical animal study	↑	Murine CLP model	Anti-LAG-3 antibody	(46)
PD-1/PD-L1	Immune checkpoint	T cells	Promotes T cell proliferation and inhibits T cell exhaustion	Preclinical animal study	↑	Murine CLP model	(D)PPA-1	(164)
TIGIT	Immune checkpoint	CD4+T cells	Promotes the proliferation and activation of CD4+ T cells	Preclinical animal study	–	Murine CLP model	Anti-TIGIT antibody	(61)
VISTA	Immune checkpoint	T cells	Inhibits T cell apoptosis and mitigates the inflammatory response	Preclinical animal study	↑	Murine CLP model	Anti-VISTA antibody (MH5A)	(65)
β1-Adrenergic Receptor	Receptor signaling	T cells	Promotes the proliferation of CD4+ T cells and suppresses the function of Treg cells	Preclinical animal study	–	Murine CLP model	esmolol	(91)
Mitochondria	Organelle dysfunction	T cells	Restores T cells’ mitochondrial function and promotes their proliferation, while reducing Tregs and MDSCs expansion	Translational study (human + murine model)	–	Human sepsis cohort and murine CLP model	citrulline	(165)

This table provides a comprehensive overview of therapeutic targets for sepsis-induced lymphocyte suppression. It details the target name, its mechanistic category, cell types affected, functions, research stages, survival rates, models used, intervention, and references for further reading. In the table, “↑” denotes improved survival with therapy, while “-” implies that it is not mentioned in the references. Evidence levels range from mechanistic *in vitro* studies to early-phase clinical investigations; most targets remain at the preclinical stage.

## 5 Conclusions and prospects

In summary, the mechanisms underlying lymphocyte suppression in sepsis have been comprehensively summarized through various pathways, including cell membrane receptors, organelles, exosomes, and non-coding RNAs. However, these mechanisms are intricately interconnected and interdependent, with each component not existing in isolation but rather interacting synergistically to collectively drive the progression of lymphocyte suppression. Despite advances, current understanding remains limited. Future investigations should integrate multi-omics technologies—such as genomics, transcriptomics, proteomics, metabolomics, and AI-driven data integration—to comprehensively unravel the complex regulatory networks governing lymphocyte suppression in sepsis. Such efforts may facilitate for novel strategies to develop individualized and precise immunomodulatory therapies.
